# Supplementary material for: The inherent flexibility of receptor binding domains in SARS-CoV-2 spike protein
Source: eLife. 2022 Mar 24;11:e75720. doi: 10.7554/eLife.75720 (PMC8963885; doi:10.7554/eLife.75720)
Supplement: Supplementary file 1. — (A) Cryo-electron microscopy (cryo-EM) structures used in the principal component analysis (PCA). (B) Definition of protomer coarse-grained particles representing rigid domains for PCA. (C) List of clusters for gREST_Down, gREST_Up, and gREST_Down w/o glycan simulations. (D) The receptor binding domain (RBD) interface cryptic pockets predicted by P2Rank. (E) List of the top-ranked molecules from the virtual screening of 2115 FDA approved drugs to RBD interface in I2a, I3a, and I3b intermediate structures. (F) Nilotinib binding energy to I2a, I3a, and I3b intermediates. [file elife-75720-supp1.docx]

**Supplementary file 1A**

Cryo-EM structures used in the PCA analysis.

“Form” is determined by the number of chains which take Down form of RBD in S-protein (3: Down, 2: 1UP, 1: 2UP, 0: 3Up, respectively). The ‘Down’ or ‘Up’ form of protomer is determined from PCA of protomers. “Different protein” means whether there are protein chain(s) other than S-protein (uniprot: P0DTC2) in Cryo-EM or X-ray structures.

| **PDB** | **Form** | **Different protein** |
| --- | --- | --- |
| 7DWX | 1UP, 1UP | Yes |
| 7JJJ | Down, Down | No |
| 6VXX | Down | No |
| 6WPS | Down | Yes |
| 6X29 | Down | No |
| 6X2C | Down | No |
| 6X6P | Down | No |
| 6X79 | Down | No |
| 6XEY | Down | Yes |
| 6XF5 | Down | No |
| 6XLU | Down | No |
| 6XM5 | Down | No |
| 6XR8 | Down | No |
| 6ZB4 | Down | No |
| 6ZB5 | Down | No |
| 6ZGE | Down | No |
| 6ZGI | Down | No |
| 6ZOX | Down | No |
| 6ZOY | Down | No |
| 6ZOZ | Down | No |
| 6ZP0 | Down | No |
| 6ZP1 | Down | No |
| 6ZP2 | Down | No |
| 6ZWV | Down | No |
| 7A4N | Down | No |
| 7BNM | Down | No |
| 7CAB | Down | No |
| 7DDD | Down | No |
| 7DF3 | Down | No |
| 7DWY | Down | No |
| 7E7B | Down | No |
| 7E7D | Down | No |
| 7JJI | Down | No |
| 7JV6 | Down | Yes |
| 7JWY | Down | No |
| 7K43 | Down | Yes |
| 7K8S | Down | Yes |
| 7K90 | Down | Yes |
| 7KDG | Down | No |
| 7KDI | Down | No |
| 7KDK | Down | No |
| 7KE4 | Down | No |
| 7KE6 | Down | No |
| 7KE7 | Down | No |
| 7KE8 | Down | No |
| 7KKK | Down | Yes |
| 7KKL | Down | Yes |
| 7KRQ | Down | No |
| 7L02 | Down | Yes |
| 7L06 | Down | Yes |
| 7L09 | Down | Yes |
| 7L2D | Down | Yes |
| 7L2E | Down | Yes |
| 7L2F | Down | Yes |
| 7L56 | Down | Yes |
| 7L57 | Down | Yes |
| 7L7K | Down | No |
| 7LAB | Down | Yes |
| 7LCN | Down | Yes |
| 7LS9 | Down | Yes |
| 7LWI | Down | No |
| 7LWJ | Down | No |
| 7LWK | Down | No |
| 7LWL | Down | No |
| 7LWS | Down | No |
| 7LXY | Down | Yes |
| 7LXZ | Down | Yes |
| 7LY2 | Down | Yes |
| 7LYL | Down | No |
| 7LYM | Down | No |
| 7M0J | Down | No |
| 7M6E | Down | Yes |
| 7MKL | Down | Yes |
| 7N01 | Down | Yes |
| 7N1T | Down | No |
| 7N1U | Down | No |
| 7N8H | Down | Yes |
| 7ND7 | Down | Yes |
| 7ND8 | Down | Yes |
| 7NDA | Down | Yes |
| 7NDC | Down | Yes |
| 7NT9 | Down | No |
| 7OAN | Down | Yes |
| 7R8N | Down | Yes |
| 7R8O | Down | Yes |
| 7RW2 | Down | Yes |
| 6VSB | 1UP | No |
| 6VYB | 1UP | No |
| 6WPT | 1UP | Yes |
| 6X2A | 1UP | No |
| 6XF6 | 1UP | No |
| 6XKL | 1UP | No |
| 6XM0 | 1UP | No |
| 6XM3 | 1UP | No |
| 6XM4 | 1UP | No |
| 6Z43 | 1UP | Yes |
| 6Z97 | 1UP | No |
| 6ZGG | 1UP | No |
| 6ZHD | 1UP | Yes |
| 6ZP7 | 1UP | No |
| 6ZXN | 1UP | Yes |
| 7A25 | 1UP | Yes |
| 7A94 | 1UP | Yes |
| 7AD1 | 1UP | No |
| 7AKD | 1UP | Yes |
| 7BNN | 1UP | No |
| 7BYR | 1UP | Yes |
| 7C2L | 1UP | Yes |
| 7CAC | 1UP | Yes |
| 7CHH | 1UP | Yes |
| 7CN9 | 1UP | No |
| 7CWM | 1UP | Yes |
| 7CZX | 1UP | Yes |
| 7D03 | 1UP | Yes |
| 7D0B | 1UP | Yes |
| 7DD8 | 1UP | Yes |
| 7DDN | 1UP | No |
| 7DF4 | 1UP | Yes |
| 7DK3 | 1UP | No |
| 7DK5 | 1UP | Yes |
| 7DWZ | 1UP | No |
| 7DX0 | 1UP | No |
| 7DX1 | 1UP | No |
| 7DX2 | 1UP | No |
| 7DX3 | 1UP | No |
| 7DX5 | 1UP | Yes |
| 7DX7 | 1UP | Yes |
| 7DZW | 1UP | No |
| 7DZX | 1UP | Yes |
| 7DZY | 1UP | Yes |
| 7EAZ | 1UP | No |
| 7EB0 | 1UP | No |
| 7EB3 | 1UP | No |
| 7EDF | 1UP | No |
| 7EDG | 1UP | No |
| 7JV4 | 1UP | Yes |
| 7K8T | 1UP | Yes |
| 7K8V | 1UP | Yes |
| 7K8W | 1UP | Yes |
| 7K8X | 1UP | Yes |
| 7K8Z | 1UP | Yes |
| 7KDH | 1UP | No |
| 7KDJ | 1UP | No |
| 7KDL | 1UP | No |
| 7KE9 | 1UP | No |
| 7KEA | 1UP | No |
| 7KEB | 1UP | No |
| 7KEC | 1UP | No |
| 7KJ2 | 1UP | Yes |
| 7KJ5 | 1UP | No |
| 7KNB | 1UP | Yes |
| 7KNE | 1UP | Yes |
| 7KRR | 1UP | No |
| 7KRS | 1UP | No |
| 7KS9 | 1UP | Yes |
| 7L58 | 1UP | Yes |
| 7LJR | 1UP | Yes |
| 7LQV | 1UP | Yes |
| 7LQW | 1UP | Yes |
| 7LWM | 1UP | No |
| 7LWN | 1UP | No |
| 7LWO | 1UP | No |
| 7LWT | 1UP | No |
| 7LWU | 1UP | No |
| 7LWV | 1UP | No |
| 7LWW | 1UP | No |
| 7LYN | 1UP | No |
| 7LYO | 1UP | No |
| 7LYP | 1UP | No |
| 7LYQ | 1UP | No |
| 7M6G | 1UP | Yes |
| 7M6H | 1UP | Yes |
| 7M6I | 1UP | Yes |
| 7M8K | 1UP | No |
| 7MJG | 1UP | No |
| 7MJH | 1UP | Yes |
| 7N0G | 1UP | Yes |
| 7N1Q | 1UP | No |
| 7N1V | 1UP | No |
| 7N1W | 1UP | No |
| 7N1X | 1UP | No |
| 7N9B | 1UP | Yes |
| 7ND3 | 1UP | Yes |
| 7ND5 | 1UP | Yes |
| 7ND6 | 1UP | Yes |
| 7ND9 | 1UP | Yes |
| 7NDB | 1UP | Yes |
| 7NDD | 1UP | Yes |
| 7NTA | 1UP | No |
| 7NTC | 1UP | Yes |
| 7P7B | 1UP | No |
| 7R8M | 1UP | Yes |
| 6X2B | 2UP | No |
| 6XCM | 2UP | Yes |
| 7A29 | 2UP | Yes |
| 7A93 | 2UP | No |
| 7A95 | 2UP | Yes |
| 7A96 | 2UP | Yes |
| 7A97 | 2UP | Yes |
| 7BNO | 2UP | No |
| 7CAI | 2UP | Yes |
| 7CWL | 2UP | Yes |
| 7CWT | 2UP | Yes |
| 7CYP | 2UP | Yes |
| 7CZP | 2UP | Yes |
| 7CZQ | 2UP | Yes |
| 7CZR | 2UP | Yes |
| 7CZT | 2UP | Yes |
| 7CZU | 2UP | Yes |
| 7CZW | 2UP | Yes |
| 7CZY | 2UP | Yes |
| 7D00 | 2UP | Yes |
| 7D0C | 2UP | Yes |
| 7D0D | 2UP | Yes |
| 7DD2 | 2UP | Yes |
| 7DK4 | 2UP | Yes |
| 7DK6 | 2UP | Yes |
| 7DX6 | 2UP | Yes |
| 7DX8 | 2UP | Yes |
| 7EB4 | 2UP | No |
| 7EB5 | 2UP | No |
| 7EDI | 2UP | No |
| 7JWB | 2UP | Yes |
| 7JZL | 2UP | Yes |
| 7JZN | 2UP | Yes |
| 7K8U | 2UP | Yes |
| 7K8Y | 2UP | Yes |
| 7KJ3 | 2UP | Yes |
| 7KL9 | 2UP | Yes |
| 7KMK | 2UP | Yes |
| 7KMZ | 2UP | Yes |
| 7KNH | 2UP | Yes |
| 7KQB | 2UP | Yes |
| 7KXK | 2UP | Yes |
| 7LWP | 2UP | No |
| 7LYK | 2UP | No |
| 7MJJ | 2UP | Yes |
| 7MJM | 2UP | Yes |
| 7MM0 | 2UP | Yes |
| 7MY3 | 2UP | Yes |
| 7N0H | 2UP | Yes |
| 7N1Y | 2UP | No |
| 7P78 | 2UP | Yes |
| 7P79 | 2UP | Yes |
| 6XCN | 3UP | Yes |
| 6ZDH | 3UP | Yes |
| 7A98 | 3UP | Yes |
| 7B18 | 3UP | Yes |
| 7CAK | 3UP | Yes |
| 7CT5 | 3UP | Yes |
| 7CWN | 3UP | Yes |
| 7CWS | 3UP | Yes |
| 7CWU | 3UP | Yes |
| 7CZS | 3UP | Yes |
| 7CZV | 3UP | Yes |
| 7CZZ | 3UP | Yes |
| 7DCC | 3UP | Yes |
| 7DCX | 3UP | Yes |
| 7DK7 | 3UP | Yes |
| 7DX9 | 3UP | Yes |
| 7E3K | 3UP | Yes |
| 7E3L | 3UP | Yes |
| 7E8C | 3UP | Yes |
| 7EDJ | 3UP | Yes |
| 7EH5 | 3UP | Yes |
| 7EJ4 | 3UP | Yes |
| 7EJ5 | 3UP | Yes |
| 7JVC | 3UP | Yes |
| 7JW0 | 3UP | Yes |
| 7K4N | 3UP | Yes |
| 7KJ4 | 3UP | Yes |
| 7KML | 3UP | Yes |
| 7KMS | 3UP | Yes |
| 7KNI | 3UP | Yes |
| 7KQE | 3UP | Yes |
| 7KSG | 3UP | Yes |
| 7KXJ | 3UP | Yes |
| 7LD1 | 3UP | Yes |
| 7LRT | 3UP | Yes |
| 7M6F | 3UP | Yes |
| 7MJK | 3UP | Yes |
| 7MY2 | 3UP | Yes |
| 7N9T | 3UP | Yes |
| 7ND4 | 3UP | Yes |
| 7P77 | 3UP | Yes |
| 7RA8 | 3UP | Yes |
| 7RKV | 3UP | Yes |
| 6ZGH | 2 chains | No |
| 6ZOW | 2 chains | No |
| 6ZP5 | 2 chains | No |
| 7EDH | 2 chains | No |
| 7L3N | 2 chains | Yes |
| 7LWQ | 2 chains | No |
| 7MTC | 2 chains | No |
| 7MTE | 2 chains | No |
| 7N9C | 2 chains | Yes |
| 7N9E | 2 chains | Yes |
| 7P7A | 2 chains | Yes |
| 7LAA | 1 chain | Yes |
| 7LSS | 1 chain | Yes |

**Supplementary file 1B**

Definition of protomer coarse-grained particles representing rigid domains for PCA.

| **Rigid domains** | **Residue numbers** |
| --- | --- |
| NTD | 27-43, 54-271 |
| NTD-b | 116-129, 169-172 |
| RBD | 330-443, 503-528 |
| RBD-h | 403-410 |
| NTD’ | 44-53,272-293 |
| SD1 | 323-329, 529-590 |
| SD2 | 294-322, 591-696 |
| S2-b | 717-727, 1047-1071 |
| CD | 711-716, 1072-1122 |

**Supplementary file 1C**

List of clusters for gREST_Down, gREST_Up and gREST_Down w/o glycan simulations.

| **Initial clusters** | **Refined clusters** | **Cluster name** | **Macro clusters** |
| --- | --- | --- | --- |
| **gREST_Down** | | | |
| C1 | C1 | D1_asym_ | Down_Like_ (Down_Asym_) |
| C2 | C2 | I1a | Down_Like_ (Int1) |
| C3 | C3 | D1_Sym_ | Down_Sym_ |
| C4 | C4(1) | I2a | Int2 |
| C5 | C5(1) | I1b | Down_Like_ (Int1) |
| C6 | C6 | I3b | Int3 |
| C7 | C7(1) | D2_asym_ | Down_Like_ (Down_Asym_) |
| C8 | C8 | D2_Sym_ | Down_Sym_ |
|  | C4(2) | I3a | Int3 |
|  | C5(2) | I1c | Down_Like_ (Int1) |
|  | C7(2) | I2b | Int2 |
|  | C7(3) | I2c | Int2 |
|  | C7(4) | 1U_L_ | 1Up |
| **gREST_Up** | | | |
| C1 | C1 | 1U_O_ | 1Up |
| C2 | C2 | 1Ub | 1Up (1U) |
| C3 | C3(1) | 1Ue | 1Up (1U) |
| C4 | C4(1) | 1Uc | 1Up (1U) |
| C5 | C5(1) | 2Ua_L_ | 1Up (2U_L_) |
| C6 | C6 | 1Uf | 1Up (1U) |
| C7 | C7(1) | 1Uh | 1Up (1U) |
| C8 | C8 | 1Ua | 1Up (1U) |
|  | C3(2) | 1Ud | 1Up (1U) |
|  | C4(2) | 1Ug | 1Up (1U) |
|  | C5(2) | 2Ub_L_ | 1Up (2U_L_) |
|  | C7(2) | 1Uj | 1Up (1U) |
|  | C7(3) | 1Ui | 1Up (1U) |
| **gREST_Down w/o glycan** | | | |
| C1 | C1(1) | 1Ua |  |
| C2 | C2 | I1b |  |
| C3 | C3(1) | I2a |  |
| C4 | C4(1) | D3 |  |
| C5 | C5 | D1 |  |
| C6 | C6 | I1a |  |
| C7 | C7 | D4 |  |
| C8 | C8(1) | D2 |  |
|  | C1(2) | 1Ub |  |
|  | C3(2) | I2b |  |
|  | C4(2) | 2Ub_L_ |  |
|  | C8(2) | 2Ua_L_ |  |

**Supplementary file 1D**

The RBD interface cryptic pockets predicted by P2Rank^30^.

| **Intermediate** | **Rank** | **Score** | **Probability** | **Residue ids** |
| --- | --- | --- | --- | --- |
| **I2a** | 3  (Pocket_1) | 113.9 | 0.994 | A_403 A_405 A_406 A_408 A_409 A_414 A_415 A_416 A_417 A_420 A_421 A_424 A_455 A_456 A_460 A_473 A_474 A_475 A_476 A_477 A_478 A_479 A_489 B_335 B_336 B_338 B_339 B_342 B_344 B_345 B_367 B_368 B_372 B_373 B_374 B_375 B_376 B_378 B_380 B_403 B_404 B_405 B_406 B_407 B_408 B_409 B_410 B_411 B_414 B_415 B_416 B_417 B_420 B_421 B_433 B_435 B_436 B_437 B_438 B_439 B_440 B_441 B_449 B_453 B_455 B_493 B_494 B_495 B_496 B_498 B_499 B_500 B_501 B_502 B_503 B_504 B_505 B_506 B_508 B_509 C_368 C_369 C_371 C_372 C_374 C_375 C_376 C_377 C_378 C_379 C_380 C_382 C_384 C_403 C_404 C_405 C_407 C_408 C_411 C_414 C_437 C_501 C_502 C_503 C_504 C_505 C_506 C_508 |
|  | 8  (Pocket_2) | 37.0 | 0.896 | A_375 A_376 A_378 A_380 A_404 A_405 A_407 A_408 A_411 A_412 A_414 A_503 A_504 A_508 C_403 C_406 C_409 C_416 C_417 C_418 C_453 C_455 C_493 C_494 C_495 C_496 C_498 C_500 C_501 C_505 |
| **I3a** | 8  (Pocket_1) | 49.6 | 0.942 | A_403 A_405 A_406 A_408 A_409 A_413 A_414 A_415 A_416 A_417 A_418 A_420 A_421 A_424 A_427 A_453 A_455 A_456 A_459 A_460 A_461 A_463 A_505 B_375 B_376 B_377 B_403 B_404 B_405 B_406 B_407 B_408 B_500 B_501 B_502 B_503 B_504 B_505 B_508 C_375 C_376 C_404 C_405 C_408 C_502 C_503 C_504 C_505 C_508 |
|  | 9  (Pocket_2) | 38.7 | 0.908 | A_375 A_376 A_378 A_380 A_404 A_405 A_407 A_408 A_411 A_412 A_414 A_502 A_503 A_504 A_505 A_508 C_403 C_406 C_409 C_415 C_416 C_417 C_418 C_449 C_453 C_455 C_493 C_494 C_495 C_496 C_498 C_500 C_501 C_505 |
| **I3b** | 5  (Pocket_2) | 37.8 | 0.904 | A_371 A_374 A_375 A_376 A_377 A_378 A_379 A_380 A_381 A_404 A_405 A_407 A_408 A_409 A_411 A_412 A_414 A_433 A_437 A_439 A_499 A_501 A_502 A_503 A_506 A_508 C_403 C_405 C_406 C_408 C_409 C_414 C_417 C_453 C_455 C_493 C_494 C_495 C_496 C_498 C_500 C_501 C_503 C_504 C_505 |
|  | 12  (Pocket_1) | 26.2 | 0.815 | A_420 A_421 A_455 A_456 A_457 A_460 A_473 A_475 A_476 A_487 A_489 B_375 B_376 B_378 B_407 B_408 B_433 B_437 B_438 B_439 B_441 B_502 B_504 B_505 B_506 B_508 |
|  | 16 | 21.6 | 0.747 | B_415 B_416 B_417 B_420 B_421 B_455 B_456 B_457 B_458 B_460 B_473 B_474 B_475 B_476 B_477 B_478 B_487 B_489 B_490 B_493 C_367 C_369 C_370 C_371 C_372 C_373 C_374 C_375 C_376 C_377 C_384 |

**Supplementary file 1E**

List of the top ranked molecules from the virtual screening of 2115 FDA approved drugs to RBD interface in I2a, I3a and I3b intermediate structures.

| **Rank** | **ZINC ID** | **Name** | **I2a^*^** | **I3a*** | **I3b*** |
| --- | --- | --- | --- | --- | --- |
| 1 | ZINC000001612996 | Irinotecan | **−**11.9 | **−**9.5 | **−**9.4 |
| 2 | ZINC000052955754 | Ergotamine | **−**11.3 | **−**9.1 | **−**9.3 |
| 3 | ZINC000003978005 | Dihydroergotamine | **−**11.1 | **−**9.9 | **−**9.4 |
| 4 | ZINC000169289767 | Trypan Blue | **−**11.1 | **−**10.0 | **−**10.1 |
| **5** | **ZINC000006716957** | Nilotinib | **−10.9** | **−9.6** | **−9.5** |
| **6** | **ZINC000006716957** | Nilotinib | **−10.8** | **−10.0** | **−9.4** |
| 7 | ZINC000003978005 | Dihydroergotamine | **−**10.8 | **−**9.8 | **−**9.8 |
| 8 | ZINC000003932831 | Avodart | **−**10.8 | **−**9.3 | **−**9.1 |
| 9 | ZINC000003978005 | Dihydroergotamine | **−**10.7 | **−**10.4 | **−**10.4 |
| 10 | ZINC000052955754 | Ergotamine | **−**10.6 | **−**9.8 | **−**9.7 |
| 11 | ZINC000052955754 | Ergotamine | **−**10.5 | **−**9.7 | **−**9.7 |
| 12 | ZINC000053683151 | Bromocriptine | **−**10.4 | **−**9.8 | **−**9.0 |
| 13 | ZINC000064033452 | Lumacaftor | **−**10.4 | **−**9.9 | **−**9.3 |
| 14 | ZINC000011679756 | Eltrombopag | **−**10.3 | **−**9.2 | **−**10.0 |
| 15 | ZINC000036701290 | Ponatinib | **−**10.3 | **−**9.6 | **−**9.5 |
| 16 | ZINC000084668739 | Lifitegrast | **−**10.2 | **−**10.1 | **−**9.3 |
| 17 | ZINC000003927822 | Lurasidone | **−**10.1 | **−**9.0 | **−**9.4 |
| 19 | ZINC000164528615 | Glecaprevir | **−**10.1 | **−**9.6 | **−**9.0 |
| 20 | ZINC000100378061 | Naldemedine | **−**10.0 | **−**9.8 | **−**9.0 |

Molecules are ranked based on binding energy to I2a while binding energy to other intermediates (I3a and I3b) are also shown.

* Binding energy in kcal mol^-1^.

Rank 5 and 6, shown in bold, represent Nilotinib.

**Supplementary file 1F**

Nilotinib binding energy to I2a, I3a and I3b intermediates.

|  | **I2a** | **I3a** | **I3b** |
| --- | --- | --- | --- |
| **Mode** | **Affinity (kcal mol^-1^)** | | |
| **1** | **−**10.9 | **−**9.6 | **−**9.5 |
| **2** | **−**10.9 | **−**9.6 | **−**9.4 |
| **3** | **−**10.8 | **−**9.5 | **−**9.3 |
| **4** | **−**10.8 | **−**9.4 | **−**9.2 |
| **5** | **−**10.6 | **−**9.3 | **−**9.2 |
| **6** | **−**10.5 | **−**9.2 | **−**9.2 |
| **7** | **−**10.4 | **−**9.0 | **−**9.2 |
| **8** | **−**10.4 | **−**8.8 | **−**9.1 |
| **9** | **−**10.3 | **−**8.7 | **−**9.1 |
